# Identification and Verification of a 17 Immune-Related Gene Pair Prognostic Signature for Colon Cancer

**DOI:** 10.1155/2021/6057948

**Published:** 2021-05-22

**Authors:** Qianshi Zhang, Zhen Feng, Yongnian Zhang, Shasha Shi, Yu Zhang, Shuangyi Ren

**Affiliations:** ^1^Departments of Gastrointestinal Surgery, The Second Affiliated Hospital of Dalian Medical University, Dalian 116023, China; ^2^Departments of Ultrasound, The Second Affiliated Hospital of Dalian Medical University, Dalian 116023, China; ^3^Departments of Clinical Laboratory, The Second Affiliated Hospital of Dalian Medical University, Dalian 116023, China

## Abstract

**Background:**

Colon cancer (CC) is a malignant tumor with a high incidence and poor prognosis. Accumulating evidence shows that the immune signature plays an important role in the tumorigenesis, progression, and prognosis of CC. Our study is aimed at establishing a novel robust immune-related gene pair signature for predicting the prognosis of CC.

**Methods:**

Gene expression profiles and corresponding clinical information are obtained from two public data sets: The Cancer Genome Atlas (TCGA) and Gene Expression Omnibus (GEO, GSE39582). We screened out immune-related gene pairs (IRGPs) associated with prognosis in the discovery cohort. Lasso-Cox proportional hazard regression was used to develop the best prognostic signature model. According to this, the patients in the validation cohort were divided into high immune-risk group and low immune-risk group, and the prediction ability of the signature model was verified by survival analysis and independent prognostic analysis.

**Results:**

A total of 17 IRGPs composed of 26 IRGs were used to construct a prognostic-related risk scoring model. This model accurately predicted the prognosis of CC patients, and the patients in the high immune-risk group indicated poor prognosis in the discovery cohort and validation cohort. Besides, whether in univariate or multivariate analysis, the IRGP signature was an independent prognostic factor. T cell CD4 memory resting in the low-risk group was significantly higher than that in the high-risk group. Functional analysis showed that the biological processes of the low-risk group included “TCA cycle” and “RNA degradation,” while the high-risk group was enriched in the “CAMs” and “focal adhesion” pathways.

**Conclusion:**

We have successfully established a signature model composed of 17 IRGPs, which provides a novel idea to predict the prognosis of CC patients.

## 1. Introduction

The incidence of colorectal cancer ranks third but second in terms of mortality around the world, and colon cancer (CC) accounts for seventy percent of colorectal cancer [1, 2]. At present, the treatment strategy is still based on surgery and supplemented by radiotherapy and chemotherapy. However, the overall five-year survival rate of CC is still only sixty-three percent [1], which is mainly due to the lack of early screening [3, 4] and the formation of resistance to postoperative standard chemotherapy drugs [5–7].

In recent years, targeted therapies such as the application of drugs targeting VEGF or EGFR [8], and immunotherapy [9–12] can be used to treat some patients with advanced CC. It should be noted that patients with the same clinical characteristics, pathological types, and treatment get different prognostic results, indicating that the innate genetic heterogeneity of patients has a great impact on clinical and molecular diversity [13].

Besides, the treatment of colorectal cancer is becoming more and more individualized and differentiated, so CC patients need to establish a clinical prognostic model based on the survival rate of patients.

Immunotherapy is the use of drugs to help a person's own immune system better recognize and destroy cancer cells, which has played an important role in the treatment of CC in the past few years [14]. CC cells prevent the immune system from attacking them by acting on “checkpoint” on immune cells if patients whose CC cells tested positive for specific gene changes, drugs called “immune checkpoint inhibitors” can be used [15, 16]. For example, the application of PD-1 [14, 15] inhibitors and CTLA-4 [14, 17] inhibitors in the treatment of CC has achieved certain results, so immunotherapy has received more and more attention.

The interaction between CC immune genes and their value in the prognosis of CC remains to be further studied. In the present study, through retrospective analysis of the public dataset, the CC immune-related genes were screened, and on the basis of what the immune-related gene pair model was built, the validity and accuracy of its prognostic features were verified.

## 2. Material and Methods

### 2.1. Data Collection and Processing

The transcriptome profiling data was downloaded from The Cancer Genome Atlas (TCGA-COAD, https://portal.gdc.cancer.gov/) treated as the discovery cohort (*n* = 452), and the validation cohort was obtained in the form of the microarray from Gene Expression Omnibus (GSE39582, *n* = 585, https://www.ncbi.nlm.nih.gov/geo/).

The gene expression profiles (GEPs) and the corresponding clinical data of the two datasets were gained and processed, respectively. In addition, the normal samples were removed, and only the tumor samples with complete survival information were retained for further analysis.

For the discovery cohort, the collection of transcriptome data, the conversion of Ensembl IDs, and the extraction of relevant clinical data were all done by performing Strawberry Perl (5.30.11). For the validation cohort, converting the probe matrix into the gene matrix was also completed by Perl.

### 2.2. Immune-Related Gene Expression Data

A list of 2498 immune genes and their action categories was retrieved from ImmPort (https://www.immport.org/shared/home) visited on March 20, 2020. Referring to the list, we gained the expression data of immune-related genes (IRGs) from the transcription matrix of discovery cohort and validation, respectively.

### 2.3. Prognosis-Related Immune Pairs

The IRGPs and clinical data were analyzed jointly. In our study, the IRGPs were measured on the platform with high variability, which was determined by the median absolute deviation [7] >0.5.

If the expression level of the first IRG is lower than the second one, the score is 0; otherwise, the score is 1. If more than 80% of the samples in the discovery cohort were score 1 or 0, the samples were filtered out, and the remaining IRGPs were left as the initial candidate prognosis-related immune gene pairs (*P* < 0.05).

### 2.4. Construction of IRGP Signature Model

After using Lasso-Cox proportional risk regression (1000 simulation iterations), we got the stable IRGPs to construct the final prognosis model (“glmnet” package, version: 3.0-2).

The model signature was presented as the risk score = (CoefficientIRGP_1_ × ScoreIRGP_1_) + (CoefficientIRGP_2_ × ScoreIRGP_2_) + ⋯+(CoefficientIRGP_*n*_ × ScoreIRGP_*n*_).

We found the optimal cut-off value through the ROC curve (“survival ROC” package), that is, the point with the greatest sum of sensitivity and specificity. Samples with a higher risk score than the cut-off value were classified as a high-risk group, and vice versa.

### 2.5. Validation of IRGP Signature

To verify the accuracy of patients' prognoses stratified by the IRGP signature, we used survival analysis and independent prognostic analysis to see whether there was statistical significance between the high-risk and low-risk groups.

### 2.6. Immune Cell Infiltration

CIBERSOFT [18] was adopted to compare the expression of immune cells between the two risk groups, which is a versatile computational method for quantifying cell fractions from bulk tissue GEPs. To quantitatively capture deconvolution confidence, CIBERSOFT calculates several quality control metrics, including a deconvolution *P* value.

TCGA-GEPs were uploaded to the CIBERSOFT portal (http://CIBERSORT.stanford.edu/), and the abundance of 22 kinds of infiltrating immune cells in each sample was calculated, including monocytes, macrophages, B cells, and T cells. The correlation of immune cell infiltration between the two risk groups was visualized by boxplot and radar chart.

### 2.7. Gene Set Enrichment Analysis (GSEA)

In order to observe the functional pathways related to the differentially expressed genes between the two risk groups, we conducted GSEA on the discovery cohort, and the KEGG (Kyoto Encyclopedia of Genes and Genomes) dataset was retrieved from GSEA datasets (“c2.cp.kegg.v7.1.symbols.gmt”, https://www.gsea-msigdb.org/). Finally, the visualization of GSEA results was shown by performing the “fgsea” package and “ggplot2” package in R software (*P* < 0.05).

### 2.8. Statistical Analysis Method

All statistical analysis was completed by R software (version 3.6.3), and data extraction and processing were realized by performing Strawberry Perl (version 5.30.2.1).

The differences among groups were compared by Student's *t*-tests or Wilcoxon rank-sum tests. The Kaplan-Meier method was used for survival analysis, and the “survival” R-package was used for the log-rank test. Cox proportional hazards regression model was used for univariable and multivariable analyses. For all the analyses, a *P* value of less than 0.05 was considered statistically significant.

## 3. Results

### 3.1. Construction and Evaluation of Prognostic IRGP Signature

This is a retrospective study of 1037 patients. The TCGA dataset (*n* = 452) and GEO dataset (*n* = 585) were used as the discovery cohort and the validation cohort, respectively (Table 1).

In the beginning, we screened 326 immune-related genes and gained 12276 immune-related gene pairs (IRGPs). After removing the less variable IRGPs and analyzing together with clinical data, Lasso-Cox proportional hazard regression was used to define the IRGP signature. Then, a total of 17 IRGPs composed of 26 IRGs were applied to construct the risk scoring model. Finally, we calculated the risk score for each patient in the discovery cohort based on the IRGP signature. The signature model was presented as a risk score = (Coefficient_IRGP1_ × Score_IRGP1_) + (Coefficient_IRGP2_ × Score_IRGP2_) + ⋯+Coefficient_IRGP17_ × Score_IRGP17_) (Table 2).

**Figure 1:**
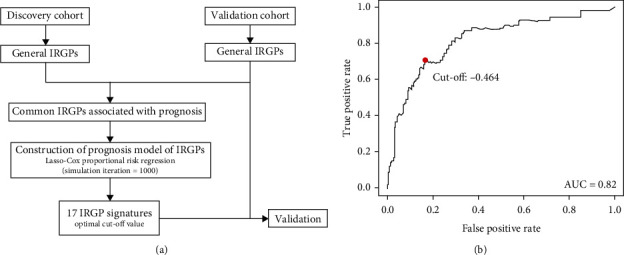
17-IRGP prognostic signature analysis flowchart (a). Time-dependent ROC analysis of the 17-IRGP prognostic signature for CC (b). Patients were stratified into the high-risk or low-risk groups based on cut-off value “-0.464”, and the AUC of the ROC curve was 0.82.

What is more, we got the cut-off value of “-0.464” by utilizing the time-dependent ROC curve analysis (Figure 1). According to the cut-off value, the patients were divided into two groups: the high-risk group and low-risk group (Table S1).

### 3.2. Verification of the IRGP Risk Model

**Figure 2:**
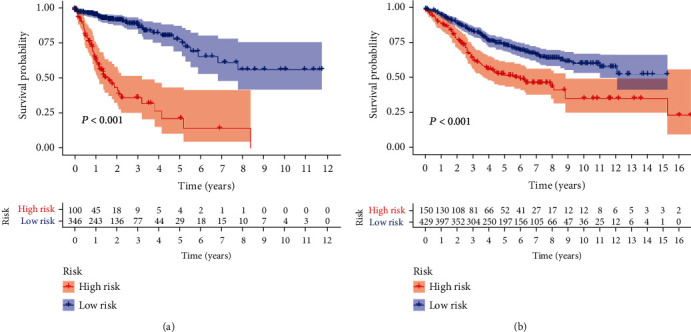
Kaplan-Meier curve of OS for CC. According to the IRGP signature, patients were stratified into two groups: the high-risk group (red) and low-risk group (blue). OS of patients in the discovering cohort (a) and validation cohort (b).

To further confirm the prognostic value of the IRGP signature in CC patients, we analyzed the survival of the high-risk and low-risk samples in the discovery cohort and the validation cohort, respectively. Both cohorts suggested that overall survival (OS) in the low-risk group was significantly better than that in the high-risk group (*P* < 0.001), and the results are shown in Figure 2.

**Figure 3:**
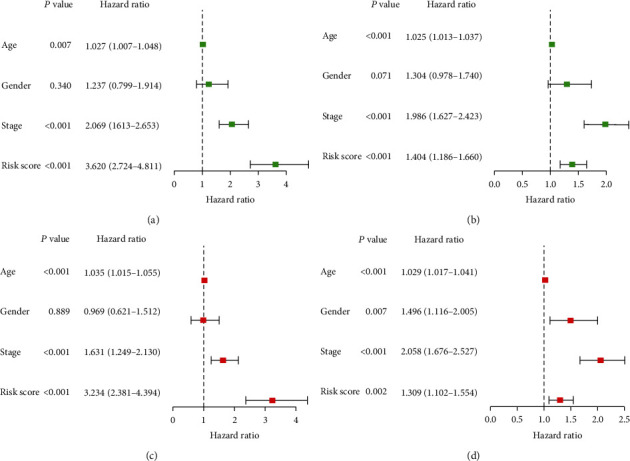
Forest plots of the hazard ratio for assessing the independent prognostic value of the IRGP signature. The univariate (a, b) and multivariate (c, d) Cox regression analysis of age, gender, stage, and risk score in the discovering cohort (a, c) and validation cohort (b, d).

For the sake of exploring whether the IRGP signature was independent of other clinical features, we performed Cox regression analysis of univariate and multivariate in two cohorts, and the results are shown in forest plots (Figure 3). Univariate Cox analysis indicated that age, tumor stage, and the IRGP risk score had a significant influence on prognosis in both two datasets (Figures 3(a) and 3(b)).

In multivariate Cox regression analysis, the risk score based on IRGP signature was an independent prognostic factor in the discovery cohort (*P* < 0.001, HR: 3.234, 95% CI: 2.381-4.394) and in the validation cohort (*P* = 0.002, HR: 1.309, 95% CI: 2.381-4.394). Besides, age and tumor stage were also statistically significant (Figures 3(c) and 3(d)).

### 3.3. Immune Cell Infiltration Correlated with IRGP Signature

**Figure 4:**
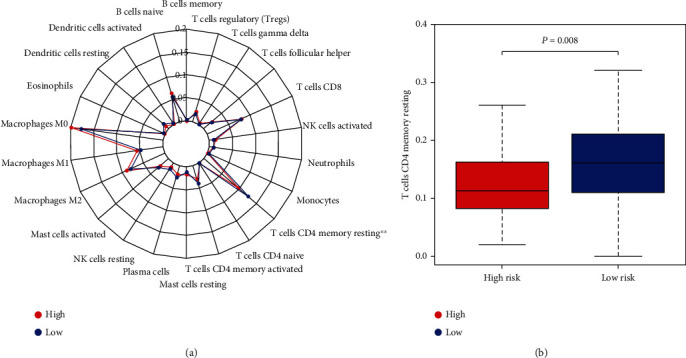
Immune cell infiltration between the two risk groups. (a) The radar plot shows the abundance of 22 immune cells estimated by CIBERSOFT within the two risk groups. (b) The significant distribution of immune cells. The expression of T cell CD4 memory resting was higher in the low-risk group (*P* = 0.008) (^∗^*P* < 0.05, ^∗∗^*P* < 0.01, and ^∗∗∗^*P* < 0.001).

We exploited CIBERSORT to estimate the abundance of 22 kinds of immune cells in each sample of two risk groups in the TCGA dataset. Radar plot showed the distribution of 22 kinds of immune cells in both the high-risk group and low-risk group (Figure 4(a)), among which T cell CD4 memory resting in the low-risk group was significantly higher than that in the high-risk group (*P* = 0.008); the graphical representation of the result is shown in Figure 4(b).

### 3.4. Biological Function Related to the IRGP Signature

**Figure 5:**
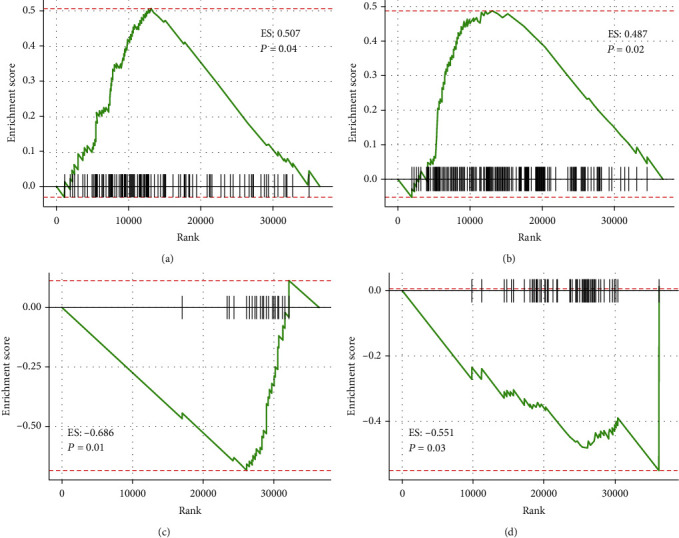
The biological function associated with the IRGP signature. GSEA shows the CC-related KEGG pathways (*P* < 0.05). The pathways enriched in the high-risk group (a, b), and the low-risk group (c, d).

To explore the biological roles of differentially expressed genes between the high-risk and low-risk immune groups, we conducted a gene set enrichment analysis on the TCGA dataset. The results showed that genes enriched in seven pathways in the high-risk group, including “cell adhesion molecules cams” and “focal adhesion,” while the expression of genes in the low-risk group was active in three pathways, including “citrate cycle TCA cycle” and “RNA degradation.” The results are shown in Figure 5 and Table S2.

## 4. Discussion

In this study, we used the k-TSP (*k*–top scoring pairs) [19] approach based on the theory of relative expression analysis (RXA) [20] to screen out the immune gene pairs related to colon cancer tissues and constructed a model to predict the prognosis of colon cancer patients according to their characteristics. This method avoids the technical deviation of standardizing gene expression profiles caused by different sequencing platforms [21], and the method can be performed by the web tool (ESurv) [22]. It shows the gene expression value through relative sequencing and pairwise comparison of the same sample, thereby enhancing the robustness of the prediction model.

Herein, the 17 immune gene pairs we screened from the TCGA dataset are composed of 26 immune genes, most of which play the role of antimicrobials, chemokines, and cytokines in the immune process. Among the 26 immune genes, RBP7 has been proved to be highly expressed as an independent biomarker of poor cancer-specific survival in early and advanced CC and is associated with CC progression [23]. The overexpression of CCL4 in CC may induce the infiltration of tumor-associated macrophages, especially the distribution of pretumor macrophages, and there was a positive correlation between plasmatic CCL4 and inflammatory mediators, which have been suggested a poor prognosis [24]. Besides, a previous study indicated that STC2 activates ERK/MEK and PI3K/AKT signaling pathways to promote colorectal tumorigenesis and epithelial-mesenchymal transition progression, and the high expression of STC2 in serum and tumor tissues is related to the low survival rate [25]. In addition, overexpression of miR-4709 promotes the proliferation and invasion of CC by downregulating NR3C2, which is an unfavorable prognostic factor [26].

Meanwhile, our model suggested CC patients with low immune risk had a better prognosis, which was subsequently verified in the validation cohort. The results showed that the content of T cell CD4 memory resting in the high immune-risk group was significantly lower than that in the low immune-risk group, which acts as the protective factor [27, 28].

The results of GSEA indicated that the differentially expressed immune-related genes between the two risk groups were enriched in the pathways: “citrate cycle TCA cycle,” “RNA degradation,” “focal adhesion,” and “cell adhesion molecules (CAMs).” Therein, the “RNA degradation” pathway can regulate the function of lncRNA GAS5 in mammalian cells [29], while GAS5 contributes to not only the susceptibility but also the lymphatic metastasis of colorectal cancer [30]. The other three pathways have been proved to be related to the tumorigenesis and progression of CC [31–33].

Although the present study merged the immune and prognostic features of colon cancer and accurately stratified the prognosis of patients, this model still has drawbacks. First of all, this study is a retrospective analysis of public datasets, which is susceptible to bias, so a large sample prospective study is needed to verify the stability of the signature model; secondly, the model based on gene-level prognostic features to predict the cost of samples is high, and the resistance of clinical promotion is relatively large.

## 5. Conclusion

In summary, we conducted a comprehensive analysis of the prognostic value of immune-related gene pairs and constructed a model that can provide a risk assessment for the treatment of colon cancer patients, so as to help them benefit from immunotherapy.

## Figures and Tables

**Table 1:** Clinical information for training and validation cohort.

	Training cohort	Validation cohort
	TCGA (*n* = 452)	GSE39582 (*n* = 585)
Age mean	67.09	66.95
Gender		
Female	214 (47%)	263 (45%)
Male	238 (53%)	322 (55%)
Stage		
I	76 (17%)	38 (7%)
II	178 (39%)	271 (46%)
III	125 (28%)	210 (36%)
IV	62 (14%)	60 (10%)
Unknown	11 (2%)	6 (1%)

**Table 2:** IRGP model information and the corresponding coefficient.

Gene pair1	Category	Gene pair 2	Category	Coefficient
CXCL14	Antimicrobials Chemokines Cytokines	BST2	Antimicrobials	-0.317773355
RBP1	Antimicrobials	STC2	Cytokines	-0.626943874
RBP7	Antimicrobials	PTGS2	Antimicrobials	0.257688669
RBP7	Antimicrobials	ARG2	Antimicrobials	0.225331574
APOD	Antimicrobials	IL17RB	Cytokine_receptors Interleukins_receptor	0.051156463
GNAI1	Antimicrobials	GRP	Cytokines	-0.241838021
CCL4	Antimicrobials Chemokines Cytokines	INHBB	Cytokines TGFb_family_member	-0.186143574
ABCC4	Antimicrobials	GRP	Cytokines	-0.283312773
ARG2	Antimicrobials	GRP	Cytokines	-0.373230092
CCR7	Antimicrobials Chemokine_receptors Cytokine_receptors	INHBB	Cytokines TGFb_family_Member	-0.306426565
CD86	Antimicrobials	IL7	Cytokines	0.278377096
C5AR1	Chemokine_receptors Cytokine_receptors	NR3C2	Cytokine_receptors	0.181534979
INHBB	Cytokines TGFb_family_member	PDGFC	Cytokines	0.49522553
STC2	Cytokines	HNF4G	Cytokine_receptors	0.289767508
IL10RA	Cytokine_receptors Interleukins_receptor	TNFRSF11A	Cytokine_receptors TNF_family_members_receptors	0.122165447
RORC	Cytokine_Receptors	PRKCQ	TCR signaling Pathway	-0.36374509
TNFRSF11A	Cytokine_receptors TNF_family_members_receptors	LCK	Natural killer_cell_cytotoxicity TCR signaling pathway	-0.34173988

## Data Availability

The data used to support the findings of this study is included within the article.

## Abbreviations

IRGP: Immune-related gene pair
CC: Colon cancer
TCGA: The Cancer Genome Atlas
GEO: Gene Expression Omnibus
OS: Overall survival
KEGG: Kyoto Encyclopedia of Genes and Genomes
GSEA: Gene set enrichment analysis
GEP: Gene expression profile.

## Supplementary Materials

Supplementary Materials Supplementary Table 1: Patients' immune risk stratification. Supplementary Table 2: GSEA.
