# Supplementary material for: Identification and Verification of a 17 Immune-Related Gene Pair Prognostic Signature for Colon Cancer
Source: Biomed Res Int. 2021 May 22;2021:6057948. doi: 10.1155/2021/6057948 (PMC8166469; doi:10.1155/2021/6057948)
Supplement: Supplementary Materials — Supplementary Table 1: Patients' immune risk stratification. Supplementary Table 2: GSEA. [file 6057948.f1.zip › Table S1 Patients' immune risk stratification.docx]

Table S1. Patients’ immune risk stratification

| **ID** | **Cohorts** | **immune risk group** | **immune risk scores** |
| --- | --- | --- | --- |
| TCGA-A6-2679  TCGA-AZ-5407  TCGA-AA-3554  TCGA-CM-6164  TCGA-AA-3517  TCGA-D5-6530  TCGA-D5-6540  TCGA-CM-6162  TCGA-AZ-4616  TCGA-CM-6676  TCGA-AA-3552  TCGA-D5-5537  TCGA-AA-3532  TCGA-AA-3542  TCGA-A6-6653  TCGA-CK-4951  TCGA-CM-5862  TCGA-NH-A8F8  TCGA-CM-5864  TCGA-AA-3660  TCGA-AY-A69D  TCGA-AZ-4308  TCGA-AA-3534  TCGA-AY-A71X  TCGA-AA-3544  TCGA-AA-3526  TCGA-CM-4751  TCGA-AA-3994  TCGA-A6-2683  TCGA-A6-6140  TCGA-CM-6168  TCGA-A6-2677  TCGA-CA-6716  TCGA-AA-3519  TCGA-QG-A5YV  TCGA-AA-3710  TCGA-CK-5916  TCGA-AD-6899  TCGA-5M-AAT4  TCGA-NH-A50U  TCGA-D5-5539  TCGA-CM-4746  TCGA-G4-6322  TCGA-AA-A00J  TCGA-AZ-6603  TCGA-AA-3548  TCGA-AA-3538  TCGA-CK-6748  TCGA-F4-6854  TCGA-CM-5868  TCGA-AA-A00L  TCGA-G4-6295  TCGA-AA-3968  TCGA-DM-A28F  TCGA-AA-3679  TCGA-DM-A28E  TCGA-AD-A5EK  TCGA-AA-3555  TCGA-D5-6923  TCGA-D5-6536  TCGA-CM-6165  TCGA-F4-6806  TCGA-A6-3809  TCGA-AA-3973  TCGA-CM-5344  TCGA-AA-A00D  TCGA-A6-3810  TCGA-AA-3862  TCGA-AA-3989  TCGA-AA-3696  TCGA-AA-3520  TCGA-AA-3666  TCGA-G4-6304  TCGA-AZ-4313  TCGA-AD-6888  TCGA-AA-3833  TCGA-CA-5256  TCGA-G4-6298  TCGA-G4-6302  TCGA-AA-A02J  TCGA-DM-A1D4  TCGA-AA-3864  TCGA-DM-A28K  TCGA-NH-A50V  TCGA-AY-6197  TCGA-A6-6141  TCGA-AA-A01G  TCGA-A6-A5ZU  TCGA-A6-3807  TCGA-AA-A02H  TCGA-CM-5348  TCGA-CK-4950  TCGA-AA-3811  TCGA-AA-A01T  TCGA-AA-A017  TCGA-AA-3506  TCGA-D5-6541  TCGA-D5-6531  TCGA-AU-6004  TCGA-DM-A1D8  TCGA-G4-6588  TCGA-F4-6855  TCGA-DM-A28A  TCGA-G4-6627  TCGA-G4-6294  TCGA-DM-A0XD  TCGA-F4-6461  TCGA-A6-6138  TCGA-CA-5797  TCGA-G4-6320  TCGA-DM-A28M  TCGA-AA-A024  TCGA-F4-6704  TCGA-AA-3979  TCGA-A6-A56B  TCGA-CM-5341  TCGA-AA-3502  TCGA-AA-A00R  TCGA-AA-A01Z  TCGA-AA-A022  TCGA-AA-3818  TCGA-D5-6929  TCGA-AA-A01V  TCGA-CA-6715  TCGA-D5-6538  TCGA-F4-6459  TCGA-CM-6680  TCGA-F4-6808  TCGA-CK-5915  TCGA-AA-3861  TCGA-QG-A5YW  TCGA-DM-A28G  TCGA-AA-3712  TCGA-CM-5860  TCGA-NH-A50T  TCGA-G4-6306  TCGA-AA-3664  TCGA-AA-A01K  TCGA-AA-3977  TCGA-AA-3530  TCGA-AA-3812  TCGA-G4-6586  TCGA-A6-5667  TCGA-AA-3692  TCGA-A6-6142  TCGA-AA-3525  TCGA-AA-3814  TCGA-DM-A1D6  TCGA-AA-3866  TCGA-AA-A03J  TCGA-D5-6927  TCGA-A6-5656  TCGA-AA-3662  TCGA-CA-5254  TCGA-AA-3837  TCGA-D5-6534  TCGA-CM-6675  TCGA-AA-A01Q  TCGA-AZ-4615  TCGA-AD-6963  TCGA-D5-6532  TCGA-AY-4071  TCGA-CM-6161  TCGA-A6-A567  TCGA-AY-4070  TCGA-CM-4744  TCGA-CK-6751  TCGA-AA-A01F  TCGA-5M-AAT6  TCGA-A6-6649  TCGA-F4-6856  TCGA-AY-5543  TCGA-AY-A54L  TCGA-AZ-6607  TCGA-AZ-6599  TCGA-AA-A01P  TCGA-AA-3715  TCGA-D5-6898  TCGA-A6-4105  TCGA-AA-3949  TCGA-AA-3939  TCGA-CK-5912  TCGA-CM-4752  TCGA-G4-6317  TCGA-CK-4952  TCGA-AA-3531  TCGA-CK-5914  TCGA-CM-6678  TCGA-CM-5861  TCGA-AA-A01I  TCGA-CM-4748  TCGA-AD-6901  TCGA-AA-3877  TCGA-4T-AA8H  TCGA-DM-A1D0  TCGA-AA-3860  TCGA-AA-A02R  TCGA-AA-3522  TCGA-AZ-5403  TCGA-AA-A01S  TCGA-NH-A6GC  TCGA-CK-6746  TCGA-AA-3815  TCGA-AA-3524  TCGA-AA-3966  TCGA-AA-A004  TCGA-CA-5255  TCGA-CK-4948  TCGA-D5-6535  TCGA-CM-6166  TCGA-AA-3673  TCGA-AA-3556  TCGA-AA-3947  TCGA-CA-6718  TCGA-AU-3779  TCGA-CM-6674  TCGA-AZ-4614  TCGA-AY-6386  TCGA-G4-6321  TCGA-F4-6805  TCGA-F4-6460  TCGA-AA-3851  TCGA-AZ-6600  TCGA-A6-5661  TCGA-G4-6310  TCGA-AM-5821  TCGA-SS-A7HO  TCGA-F4-6703  TCGA-CM-5349  TCGA-A6-2675  TCGA-F4-6570  TCGA-A6-6781  TCGA-AA-3971  TCGA-QL-A97D  TCGA-DM-A1HA  TCGA-D5-6529  TCGA-RU-A8FL  TCGA-AA-3713  TCGA-A6-5659  TCGA-AA-3982  TCGA-CM-6171  TCGA-G4-6297  TCGA-DM-A1D9  TCGA-AA-3869  TCGA-AA-A00U  TCGA-DM-A28H  TCGA-WS-AB45  TCGA-AA-3984  TCGA-AA-3680  TCGA-AD-6548  TCGA-AA-3831  TCGA-DM-A0XF  TCGA-AA-3841  TCGA-G4-6309  TCGA-CA-5796  TCGA-AA-3663  TCGA-QG-A5YX  TCGA-AA-3560  TCGA-AA-A02K  TCGA-AA-3930  TCGA-AA-3693  TCGA-A6-2680  TCGA-A6-3808  TCGA-A6-6650  TCGA-F4-6569  TCGA-AA-3846  TCGA-AA-A00F  TCGA-A6-5657  TCGA-AA-3870  TCGA-D5-6926  TCGA-G4-6628  TCGA-D5-6533  TCGA-AA-3856  TCGA-AD-6889  TCGA-A6-5666  TCGA-AA-3675  TCGA-G4-6299  TCGA-AA-3867  TCGA-DM-A1D7  TCGA-AD-A5EJ  TCGA-AD-6895  TCGA-AD-6964  TCGA-AA-3950  TCGA-D5-6932  TCGA-G4-6307  TCGA-AA-A02E  TCGA-AA-A02F  TCGA-A6-2672  TCGA-DM-A280  TCGA-CM-4743  TCGA-AA-3518  TCGA-A6-2674  TCGA-AA-3496  TCGA-AZ-6606  TCGA-AA-A00K  TCGA-CM-6169  TCGA-AA-3678  TCGA-AA-A00E  TCGA-AA-A00Q  TCGA-AA-3549  TCGA-G4-6625  TCGA-AD-5900  TCGA-A6-6651  TCGA-CK-5913  TCGA-A6-2681  TCGA-T9-A92H  TCGA-AA-3941  TCGA-NH-A8F7  TCGA-A6-A566  TCGA-AA-3561  TCGA-DM-A28C  TCGA-D5-5538  TCGA-AA-A01R  TCGA-AA-3681  TCGA-AA-A00Z  TCGA-NH-A6GB  TCGA-A6-2686  TCGA-CM-6170  TCGA-A6-2678  TCGA-AA-3514  TCGA-CM-6167  TCGA-AA-A00A  TCGA-DM-A1DB  TCGA-AA-3956  TCGA-CK-6747  TCGA-AA-3970  TCGA-A6-6780  TCGA-AD-6965  TCGA-AA-3672  TCGA-AA-3655  TCGA-AM-5820  TCGA-G4-6311  TCGA-A6-5660  TCGA-AZ-6601  TCGA-AA-3850  TCGA-D5-6920  TCGA-AA-3553  TCGA-AA-3527  TCGA-D5-7000  TCGA-NH-A5IV  TCGA-A6-5665  TCGA-CM-6163  TCGA-AA-3688  TCGA-AA-3872  TCGA-AA-3855  TCGA-D5-6930  TCGA-5M-AATE  TCGA-AA-3975  TCGA-AA-3952  TCGA-A6-2671  TCGA-A6-6654  TCGA-A6-2682  TCGA-CM-6677  TCGA-AZ-4315  TCGA-AA-3562  TCGA-AA-A01X  TCGA-DM-A0X9  TCGA-AA-3516  TCGA-AA-3845  TCGA-CM-5863  TCGA-CA-6719  TCGA-A6-6652  TCGA-AY-A8YK  TCGA-AZ-6608  TCGA-A6-2684  TCGA-AA-3543  TCGA-CK-4947  TCGA-AA-3684  TCGA-AA-3980  TCGA-AA-3529  TCGA-AA-A01C  TCGA-DM-A1HB  TCGA-AA-A00O  TCGA-A6-6648  TCGA-AA-3511  TCGA-AA-A02W  TCGA-CM-4747  TCGA-CM-6679  TCGA-3L-AA1B  TCGA-AA-3492  TCGA-AA-A03F  TCGA-AA-A02Y  TCGA-G4-6323  TCGA-CA-6717  TCGA-AA-A00N  TCGA-AA-A010  TCGA-DM-A285  TCGA-D5-5541  TCGA-G4-6314  TCGA-AZ-6598  TCGA-AA-3494  TCGA-A6-2676  TCGA-AA-A00W  TCGA-AD-6890  TCGA-A6-6782  TCGA-A6-5664  TCGA-AA-3972  TCGA-AA-3955  TCGA-D5-5540  TCGA-AA-3854  TCGA-F4-6463  TCGA-D5-6924  TCGA-AA-A02O  TCGA-AA-3852  TCGA-AA-3875  TCGA-AA-3667  TCGA-AA-A029  TCGA-NH-A6GA  TCGA-D5-6922  TCGA-A6-6137  TCGA-AA-3697  TCGA-AA-3509  TCGA-A6-5662  TCGA-AZ-4323  TCGA-AA-3510  TCGA-F4-6807  TCGA-G4-6303  TCGA-AA-3842  TCGA-DM-A288  TCGA-AA-3844  TCGA-D5-6537  TCGA-DM-A1DA  TCGA-CM-6172  TCGA-A6-2685  TCGA-AA-3685  TCGA-AA-3858  TCGA-AA-3819  TCGA-G4-6626  TCGA-D5-6928  TCGA-A6-A565  TCGA-AA-3821  TCGA-4N-A93T  TCGA-AA-3986  TCGA-DM-A282  TCGA-AA-3489  TCGA-F4-6809  TCGA-D5-6931  TCGA-D5-6539  TCGA-AZ-6605  TCGA-QG-A5Z2  TCGA-AY-6196  TCGA-G4-6293  TCGA-G4-6315  TCGA-AA-3848  TCGA-AA-3495  TCGA-A6-4107 | TCGA  TCGA  TCGA  TCGA  TCGA  TCGA  TCGA  TCGA  TCGA  TCGA  TCGA  TCGA  TCGA  TCGA  TCGA  TCGA  TCGA  TCGA  TCGA  TCGA  TCGA  TCGA  TCGA  TCGA  TCGA  TCGA  TCGA  TCGA  TCGA  TCGA  TCGA  TCGA  TCGA  TCGA  TCGA  TCGA  TCGA  TCGA  TCGA  TCGA  TCGA  TCGA  TCGA  TCGA  TCGA  TCGA  TCGA  TCGA  TCGA  TCGA  TCGA  TCGA  TCGA  TCGA  TCGA  TCGA  TCGA  TCGA  TCGA  TCGA  TCGA  TCGA  TCGA  TCGA  TCGA  TCGA  TCGA  TCGA  TCGA  TCGA  TCGA  TCGA  TCGA  TCGA  TCGA  TCGA  TCGA  TCGA  TCGA  TCGA  TCGA  TCGA  TCGA  TCGA  TCGA  TCGA  TCGA  TCGA  TCGA  TCGA  TCGA  TCGA  TCGA  TCGA  TCGA  TCGA  TCGA  TCGA  TCGA  TCGA  TCGA  TCGA  TCGA  TCGA  TCGA  TCGA  TCGA  TCGA  TCGA  TCGA  TCGA  TCGA  TCGA  TCGA  TCGA  TCGA  TCGA  TCGA  TCGA  TCGA  TCGA  TCGA  TCGA  TCGA  TCGA  TCGA  TCGA  TCGA  TCGA  TCGA  TCGA  TCGA  TCGA  TCGA  TCGA  TCGA  TCGA  TCGA  TCGA  TCGA  TCGA  TCGA  TCGA  TCGA  TCGA  TCGA  TCGA  TCGA  TCGA  TCGA  TCGA  TCGA  TCGA  TCGA  TCGA  TCGA  TCGA  TCGA  TCGA  TCGA  TCGA  TCGA  TCGA  TCGA  TCGA  TCGA  TCGA  TCGA  TCGA  TCGA  TCGA  TCGA  TCGA  TCGA  TCGA  TCGA  TCGA  TCGA  TCGA  TCGA  TCGA  TCGA  TCGA  TCGA  TCGA  TCGA  TCGA  TCGA  TCGA  TCGA  TCGA  TCGA  TCGA  TCGA  TCGA  TCGA  TCGA  TCGA  TCGA  TCGA  TCGA  TCGA  TCGA  TCGA  TCGA  TCGA  TCGA  TCGA  TCGA  TCGA  TCGA  TCGA  TCGA  TCGA  TCGA  TCGA  TCGA  TCGA  TCGA  TCGA  TCGA  TCGA  TCGA  TCGA  TCGA  TCGA  TCGA  TCGA  TCGA  TCGA  TCGA  TCGA  TCGA  TCGA  TCGA  TCGA  TCGA  TCGA  TCGA  TCGA  TCGA  TCGA  TCGA  TCGA  TCGA  TCGA  TCGA  TCGA  TCGA  TCGA  TCGA  TCGA  TCGA  TCGA  TCGA  TCGA  TCGA  TCGA  TCGA  TCGA  TCGA  TCGA  TCGA  TCGA  TCGA  TCGA  TCGA  TCGA  TCGA  TCGA  TCGA  TCGA  TCGA  TCGA  TCGA  TCGA  TCGA  TCGA  TCGA  TCGA  TCGA  TCGA  TCGA  TCGA  TCGA  TCGA  TCGA  TCGA  TCGA  TCGA  TCGA  TCGA  TCGA  TCGA  TCGA  TCGA  TCGA  TCGA  TCGA  TCGA  TCGA  TCGA  TCGA  TCGA  TCGA  TCGA  TCGA  TCGA  TCGA  TCGA  TCGA  TCGA  TCGA  TCGA  TCGA  TCGA  TCGA  TCGA  TCGA  TCGA  TCGA  TCGA  TCGA  TCGA  TCGA  TCGA  TCGA  TCGA  TCGA  TCGA  TCGA  TCGA  TCGA  TCGA  TCGA  TCGA  TCGA  TCGA  TCGA  TCGA  TCGA  TCGA  TCGA  TCGA  TCGA  TCGA  TCGA  TCGA  TCGA  TCGA  TCGA  TCGA  TCGA  TCGA  TCGA  TCGA  TCGA  TCGA  TCGA  TCGA  TCGA  TCGA  TCGA  TCGA  TCGA  TCGA  TCGA  TCGA  TCGA  TCGA  TCGA  TCGA  TCGA  TCGA  TCGA  TCGA  TCGA  TCGA  TCGA  TCGA  TCGA  TCGA  TCGA  TCGA  TCGA  TCGA  TCGA  TCGA  TCGA  TCGA  TCGA  TCGA  TCGA  TCGA  TCGA  TCGA  TCGA  TCGA  TCGA  TCGA  TCGA  TCGA  TCGA  TCGA  TCGA  TCGA  TCGA  TCGA  TCGA  TCGA  TCGA  TCGA  TCGA  TCGA  TCGA  TCGA  TCGA  TCGA  TCGA  TCGA  TCGA  TCGA  TCGA  TCGA  TCGA  TCGA  TCGA  TCGA  TCGA  TCGA  TCGA  TCGA  TCGA  TCGA  TCGA  TCGA  TCGA  TCGA  TCGA  TCGA  TCGA  TCGA  TCGA  TCGA  TCGA  TCGA | Low Immune Risk  Low Immune Risk  Low Immune Risk  Low Immune Risk  Low Immune Risk  Low Immune Risk  Low Immune Risk  Low Immune Risk  High Immune Risk  Low Immune Risk  Low Immune Risk  High Immune Risk  Low Immune Risk  Low Immune Risk  Low Immune Risk  Low Immune Risk  High Immune Risk  High Immune Risk  Low Immune Risk  Low Immune Risk  Low Immune Risk  Low Immune Risk  Low Immune Risk  Low Immune Risk  Low Immune Risk  Low Immune Risk  High Immune Risk  High Immune Risk  Low Immune Risk  Low Immune Risk  High Immune Risk  Low Immune Risk  Low Immune Risk  Low Immune Risk  Low Immune Risk  Low Immune Risk  High Immune Risk  High Immune Risk  High Immune Risk  High Immune Risk  Low Immune Risk  Low Immune Risk  Low Immune Risk  Low Immune Risk  Low Immune Risk  Low Immune Risk  Low Immune Risk  High Immune Risk  Low Immune Risk  Low Immune Risk  Low Immune Risk  Low Immune Risk  Low Immune Risk  Low Immune Risk  Low Immune Risk  Low Immune Risk  Low Immune Risk  Low Immune Risk  Low Immune Risk  Low Immune Risk  Low Immune Risk  Low Immune Risk  Low Immune Risk  Low Immune Risk  High Immune Risk  Low Immune Risk  High Immune Risk  Low Immune Risk  High Immune Risk  Low Immune Risk  Low Immune Risk  Low Immune Risk  Low Immune Risk  Low Immune Risk  Low Immune Risk  Low Immune Risk  Low Immune Risk  Low Immune Risk  High Immune Risk  Low Immune Risk  Low Immune Risk  Low Immune Risk  Low Immune Risk  Low Immune Risk  Low Immune Risk  Low Immune Risk  Low Immune Risk  High Immune Risk  Low Immune Risk  Low Immune Risk  Low Immune Risk  Low Immune Risk  Low Immune Risk  Low Immune Risk  Low Immune Risk  Low Immune Risk  High Immune Risk  High Immune Risk  Low Immune Risk  High Immune Risk  Low Immune Risk  Low Immune Risk  High Immune Risk  Low Immune Risk  Low Immune Risk  High Immune Risk  High Immune Risk  Low Immune Risk  Low Immune Risk  Low Immune Risk  Low Immune Risk  Low Immune Risk  High Immune Risk  Low Immune Risk  Low Immune Risk  Low Immune Risk  Low Immune Risk  High Immune Risk  Low Immune Risk  Low Immune Risk  High Immune Risk  High Immune Risk  Low Immune Risk  Low Immune Risk  Low Immune Risk  High Immune Risk  High Immune Risk  Low Immune Risk  Low Immune Risk  Low Immune Risk  Low Immune Risk  Low Immune Risk  Low Immune Risk  Low Immune Risk  Low Immune Risk  Low Immune Risk  Low Immune Risk  Low Immune Risk  Low Immune Risk  Low Immune Risk  Low Immune Risk  Low Immune Risk  Low Immune Risk  Low Immune Risk  High Immune Risk  Low Immune Risk  Low Immune Risk  Low Immune Risk  Low Immune Risk  Low Immune Risk  Low Immune Risk  Low Immune Risk  Low Immune Risk  Low Immune Risk  Low Immune Risk  High Immune Risk  Low Immune Risk  High Immune Risk  Low Immune Risk  Low Immune Risk  Low Immune Risk  Low Immune Risk  Low Immune Risk  High Immune Risk  High Immune Risk  Low Immune Risk  Low Immune Risk  Low Immune Risk  High Immune Risk  Low Immune Risk  Low Immune Risk  Low Immune Risk  Low Immune Risk  High Immune Risk  Low Immune Risk  High Immune Risk  High Immune Risk  Low Immune Risk  Low Immune Risk  Low Immune Risk  Low Immune Risk  Low Immune Risk  Low Immune Risk  Low Immune Risk  Low Immune Risk  Low Immune Risk  Low Immune Risk  High Immune Risk  Low Immune Risk  Low Immune Risk  Low Immune Risk  High Immune Risk  Low Immune Risk  Low Immune Risk  Low Immune Risk  Low Immune Risk  Low Immune Risk  Low Immune Risk  Low Immune Risk  Low Immune Risk  Low Immune Risk  High Immune Risk  Low Immune Risk  Low Immune Risk  High Immune Risk  Low Immune Risk  Low Immune Risk  Low Immune Risk  Low Immune Risk  High Immune Risk  Low Immune Risk  Low Immune Risk  Low Immune Risk  High Immune Risk  Low Immune Risk  Low Immune Risk  High Immune Risk  Low Immune Risk  Low Immune Risk  High Immune Risk  Low Immune Risk  Low Immune Risk  High Immune Risk  Low Immune Risk  Low Immune Risk  High Immune Risk  High Immune Risk  High Immune Risk  Low Immune Risk  Low Immune Risk  High Immune Risk  Low Immune Risk  Low Immune Risk  Low Immune Risk  Low Immune Risk  Low Immune Risk  Low Immune Risk  Low Immune Risk  Low Immune Risk  Low Immune Risk  Low Immune Risk  High Immune Risk  Low Immune Risk  Low Immune Risk  Low Immune Risk  Low Immune Risk  Low Immune Risk  Low Immune Risk  Low Immune Risk  Low Immune Risk  Low Immune Risk  Low Immune Risk  Low Immune Risk  Low Immune Risk  Low Immune Risk  Low Immune Risk  Low Immune Risk  Low Immune Risk  High Immune Risk  High Immune Risk  Low Immune Risk  Low Immune Risk  Low Immune Risk  Low Immune Risk  High Immune Risk  Low Immune Risk  Low Immune Risk  High Immune Risk  Low Immune Risk  Low Immune Risk  Low Immune Risk  Low Immune Risk  Low Immune Risk  Low Immune Risk  Low Immune Risk  Low Immune Risk  Low Immune Risk  Low Immune Risk  Low Immune Risk  Low Immune Risk  High Immune Risk  High Immune Risk  Low Immune Risk  High Immune Risk  Low Immune Risk  High Immune Risk  Low Immune Risk  Low Immune Risk  Low Immune Risk  Low Immune Risk  Low Immune Risk  High Immune Risk  High Immune Risk  High Immune Risk  Low Immune Risk  High Immune Risk  Low Immune Risk  Low Immune Risk  High Immune Risk  Low Immune Risk  Low Immune Risk  Low Immune Risk  High Immune Risk  Low Immune Risk  Low Immune Risk  Low Immune Risk  Low Immune Risk  Low Immune Risk  High Immune Risk  Low Immune Risk  Low Immune Risk  Low Immune Risk  High Immune Risk  Low Immune Risk  Low Immune Risk  Low Immune Risk  Low Immune Risk  Low Immune Risk  Low Immune Risk  Low Immune Risk  High Immune Risk  Low Immune Risk  Low Immune Risk  Low Immune Risk  Low Immune Risk  Low Immune Risk  Low Immune Risk  High Immune Risk  Low Immune Risk  Low Immune Risk  High Immune Risk  Low Immune Risk  Low Immune Risk  High Immune Risk  Low Immune Risk  Low Immune Risk  Low Immune Risk  Low Immune Risk  Low Immune Risk  High Immune Risk  Low Immune Risk  Low Immune Risk  Low Immune Risk  High Immune Risk  Low Immune Risk  Low Immune Risk  Low Immune Risk  Low Immune Risk  High Immune Risk  High Immune Risk  Low Immune Risk  Low Immune Risk  Low Immune Risk  Low Immune Risk  Low Immune Risk  Low Immune Risk  Low Immune Risk  Low Immune Risk  High Immune Risk  Low Immune Risk  High Immune Risk  Low Immune Risk  Low Immune Risk  Low Immune Risk  Low Immune Risk  Low Immune Risk  Low Immune Risk  High Immune Risk  Low Immune Risk  Low Immune Risk  Low Immune Risk  Low Immune Risk  Low Immune Risk  Low Immune Risk  Low Immune Risk  Low Immune Risk  Low Immune Risk  Low Immune Risk  Low Immune Risk  Low Immune Risk  Low Immune Risk  Low Immune Risk  Low Immune Risk  Low Immune Risk  High Immune Risk  Low Immune Risk  High Immune Risk  Low Immune Risk  High Immune Risk  High Immune Risk  Low Immune Risk  Low Immune Risk  Low Immune Risk  Low Immune Risk  High Immune Risk  Low Immune Risk  Low Immune Risk  Low Immune Risk  Low Immune Risk  Low Immune Risk  High Immune Risk  High Immune Risk  High Immune Risk  Low Immune Risk  Low Immune Risk  Low Immune Risk  Low Immune Risk  High Immune Risk  Low Immune Risk  Low Immune Risk  Low Immune Risk  Low Immune Risk  Low Immune Risk  High Immune Risk  Low Immune Risk  High Immune Risk  Low Immune Risk  Low Immune Risk  High Immune Risk  Low Immune Risk  High Immune Risk  High Immune Risk  Low Immune Risk  High Immune Risk  Low Immune Risk  Low Immune Risk  Low Immune Risk  Low Immune Risk  Low Immune Risk  High Immune Risk  Low Immune Risk  Low Immune Risk  Low Immune Risk  Low Immune Risk  High Immune Risk  High Immune Risk  Low Immune Risk  Low Immune Risk  High Immune Risk  Low Immune Risk  High Immune Risk  Low Immune Risk  Low Immune Risk  High Immune Risk  Low Immune Risk  Low Immune Risk | -0.650444034  -3.041153226  -1.646076818  -0.926886991  -1.244878703  -2.096435996  -0.77150904  -1.272778832  0.759241601  -1.684972883  -1.14549309  -0.034234577  -1.624433156  -1.728584167  -1.732690907  -0.501716718  -0.070212652  0.785099348  -1.453929005  -2.119658547  -2.040979259  -1.355201464  -1.237176581  -1.168725013  -0.952797134  -1.529933355  -0.393225811  -0.066825973  -1.296360553  -2.014169556  -0.033473975  -1.799473765  -1.744512423  -1.233206026  -0.878957505  -1.172618593  -0.395086373  0.315131631  0.281390678  0.900237648  -1.931523021  -2.72337987  -2.050464262  -0.724514245  -0.731317334  -1.225570434  -1.987193991  -0.311483244  -1.136646174  -0.667873623  -1.136646174  -0.932615463  -1.217322095  -0.544542513  -2.76277613  -1.426413682  -0.464106926  -0.966352266  -1.393018201  -0.709416581  -2.310340105  -1.05318359  -1.354153572  -0.867347451  0.484093392  -0.638441887  0.598444403  -1.863744554  0.222461415  -0.737195146  -2.263467796  -0.637583389  -0.818872819  -2.441724675  -0.561184149  -2.263467796  -1.455103325  -0.66138917  -0.373961426  -1.431923797  -0.747327723  -1.636480299  -1.584507926  -0.726600013  -2.096435996  -1.414360772  -1.082071603  -0.192208091  -0.576296443  -0.626837565  -1.620160779  -3.041153226  -0.708142863  -1.756851853  -1.304601781  -1.582055069  -0.047298839  0.711233816  -1.636523922  -0.036033356  -0.66115146  -0.51492408  -0.015904081  -1.461822303  -1.294740418  -0.168646214  0.159091292  -1.72227053  -1.390182042  -3.041153226  -0.878957505  -1.15497667  0.499171717  -2.235418326  -0.551955839  -1.861536212  -2.699413344  -0.134328156  -1.629266401  -1.251556259  0.195097841  -0.185637798  -2.053357556  -1.505901379  -1.374183035  0.206373943  -0.051839449  -2.23950113  -1.426413682  -2.783464556  -1.878143587  -1.138799749  -2.189446591  -1.816216499  -1.327838751  -2.330483526  -1.294783901  -0.664121831  -1.346756414  -1.426413682  -1.065434992  -1.294783901  -1.348714166  -1.274640619  0.061576839  -2.23950113  -1.218954936  -1.374183035  -2.217496061  -1.168813715  -1.040329848  -1.586558933  -1.594060822  -1.578875461  -0.894102032  -0.003259785  -0.710093566  0.003154644  -0.555932925  -1.622110292  -1.374183035  -2.117335822  -1.84770671  0.188625525  0.800634449  -1.117647925  -1.571285203  -1.760363071  0.321497277  -1.660461211  -1.787590864  -1.806668488  -2.053357556  -0.028499471  -0.826985131  -0.293269752  0.647279788  -0.785722887  -0.471126736  -1.323166248  -2.466721902  -1.589452227  -1.66162803  -1.608256096  -0.988357475  -0.96062131  -1.244729362  -0.22221793  -0.696222234  -1.005016393  -0.638626462  0.498625348  -2.541844892  -1.795668887  -1.795668887  -0.928627735  -0.808873503  -2.667923133  -1.574378478  -0.537217623  -0.825442076  -0.253677669  -2.096435996  -2.07246947  -0.007221407  -1.240315187  -1.870571312  -1.195841144  -1.624762543  0.035252383  -2.326183252  -3.041153226  -1.899722706  0.317270789  -0.619524224  -1.072408687  0.358569065  -2.363052888  -2.288239026  0.527414389  -0.520898103  -1.957253434  0.422974544  -2.07246947  -1.917664325  0.190312579  -0.115172758  0.189778933  -0.483332023  -1.171790552  0.415048863  -0.57642452  -2.76277613  -2.263467796  -0.590875581  -0.815813587  -0.537217623  -1.918983821  -1.477536361  -2.453930997  -1.954297277  -0.245572897  -1.820495126  -0.512767839  -1.393844319  -0.708812938  -0.57642452  -1.689569443  -3.041153226  -0.599538313  -1.9818126  -1.01504654  -0.613371313  -1.738852968  -1.426413682  -1.868929283  -2.414209352  -2.421036248  -0.444085104  0.315372068  -1.636638304  -1.496276282  -1.249341902  -2.013666809  0.378952774  -2.232674373  -1.54840912  -0.251388896  -0.850025118  -0.834701817  -1.062757294  -0.591366105  -2.577247897  -2.107782786  -1.025057244  -0.818872819  -0.629864121  -1.680434899  -0.596021093  -1.500241923  0.180967549  -0.332015321  -1.040329848  0.402239343  -2.156520682  0.879270965  -0.966986955  -1.172618593  -0.779106768  -0.931038811  -1.790009431  0.012652144  -0.201111014  -0.403155355  -1.755512025  -0.216473898  -1.19372224  -1.768242265  0.128050979  -1.090143893  -1.686287952  -1.548497822  1.300161139  -1.885302027  -1.613749666  -1.065101078  -0.979399542  -1.921639212  0.201876959  -2.251706417  -1.168725013  -0.531722792  -0.152242269  -1.276287467  -0.637119483  -0.780973374  -0.910666583  -1.74410573  -1.78845991  -0.664596559  0.095640001  -0.933759096  -1.068135684  -2.414209352  -0.512182148  -3.041153226  -1.403157348  0.037987709  -0.823481414  -1.755512025  -0.236259185  -0.894726846  -1.703548692  -0.355129114  -2.117335822  -1.918983821  -1.875497801  -0.567683965  -1.914901018  0.111237836  -2.445002775  -1.867657349  -2.107782786  -0.348192242  -1.554257481  -2.30000687  -1.582055069  -1.892004248  1.537502177  -0.062084074  -1.094893406  -1.356760785  -0.989851703  -2.323552482  -2.505087461  -1.13697156  -1.058693705  -1.886414459  -0.188869713  -0.786684699  0.108171561  -1.438289443  -2.228065778  -0.878957505  -0.776289995  -1.981097446  -3.041153226  -0.132073317  -2.392986779  -1.766858815  -1.086666191  -1.294783901  -0.538409894  -2.107782786  -2.276703644  -1.679801191  -0.922285228  -0.727899693  -1.280607095  -0.594755016  -1.393833027  -2.72337987  -3.041153226  -0.492925112  -0.167383632  -0.468950628  0.643623162  -0.809330139  -0.173434285  -0.150075281  -2.414209352  -0.471126736  -1.629216313  -1.608437629  -0.034720563  -1.384810255  -1.871822577  -3.041153226  -1.196099522  -2.456349564  -0.216800257  -0.138851189  -0.110794579  -0.973526321  -2.859618247  -1.148081665  -2.107782786  -0.458379899  -1.707240243  -1.732840247  -2.581241151  -2.808461783  -0.747073224  1.069669908  -0.791227841  -0.260276118  -2.31135366  -0.749489826  -0.149201562  -2.07246947  -0.286872221  -0.049299701  -1.931189108  -0.378351665  -1.768769044  -1.520764142  -3.041153226  -1.294783901  -0.574056927  -0.415095799  -2.72337987  -1.093111265  -2.154133275  -1.025057244  0.745177712  -0.388365126  -0.506894854  -2.558132982  -0.082422095  -3.041153226  0.146551152  -2.577247897  -1.426413682  -0.017220862  -2.07246947  -1.288647138 |
| GSM972368  GSM972356  GSM972049  GSM972155  GSM972373  GSM972144  GSM972439  GSM972210  GSM972198  GSM971992  GSM972100  GSM971994  GSM972138  GSM972279  GSM971983  GSM972142  GSM972499  GSM972301  GSM972393  GSM972124  GSM972239  GSM972029  GSM972410  GSM972384  GSM972163  GSM972178  GSM972307  GSM971972  GSM972382  GSM971974  GSM972516  GSM972333  GSM972479  GSM972299  GSM972015  GSM972122  GSM1681370  GSM972157  GSM972328  GSM972188  GSM971982  GSM972374  GSM972011  GSM972143  GSM972416  GSM971984  GSM972510  GSM972372  GSM971993  GSM972489  GSM972164  GSM972383  GSM972069  GSM972350  GSM972348  GSM972394  GSM972332  GSM972106  GSM972305  GSM972123  GSM972216  GSM972392  GSM972334  GSM972151  GSM971968  GSM972162  GSM972289  GSM971973  GSM972017  GSM971960  GSM972136  GSM972013  GSM971977  GSM972141  GSM972302  GSM1681354  GSM972335  GSM972304  GSM972196  GSM972387  GSM972395  GSM972127  GSM972358  GSM972340  GSM972366  GSM972165  GSM972509  GSM971991  GSM972381  GSM972121  GSM971997  GSM971985  GSM972518  GSM972375  GSM972153  GSM972180  GSM972176  GSM972320  GSM971971  GSM972147  GSM1681367  GSM972119  GSM972371  GSM972014  GSM972209  GSM971975  GSM972337  GSM972170  GSM972186  GSM972385  GSM972418  GSM972397  GSM972012  GSM972125  GSM972303  GSM972167  GSM972326  GSM971981  GSM971958  GSM971966  GSM972152  GSM972409  GSM972391  GSM971995  GSM971987  GSM972161  GSM972130  GSM972108  GSM972377  GSM972190  GSM972218  GSM972154  GSM1681362  GSM972059  GSM972145  GSM972331  GSM972360  GSM972346  GSM972075  GSM972419  GSM972252  GSM972230  GSM972020  GSM972261  GSM972290  GSM972118  GSM972470  GSM972521  GSM972081  GSM972208  GSM972486  GSM972467  GSM972254  GSM972425  GSM972245  GSM972109  GSM972004  GSM972219  GSM972095  GSM972452  GSM972087  GSM972040  GSM972066  GSM972430  GSM972058  GSM972461  GSM971959  GSM972490  GSM972270  GSM972408  GSM972286  GSM1681355  GSM972267  GSM972002  GSM972313  GSM972035  GSM972454  GSM972225  GSM972445  GSM972077  GSM972359  GSM972441  GSM972436  GSM972060  GSM972046  GSM972508  GSM972221  GSM972031  GSM972280  GSM972276  GSM972091  GSM972496  GSM972253  GSM972465  GSM972427  GSM972247  GSM972097  GSM972085  GSM972003  GSM972312  GSM972241  GSM972236  GSM972026  GSM972421  GSM972480  GSM972314  GSM972071  GSM972476  GSM972296  GSM972453  GSM972265  GSM972227  GSM972037  GSM972447  GSM972519  GSM972082  GSM972522  GSM972457  GSM972264  GSM972443  GSM972116  GSM972223  GSM972033  GSM972488  GSM972315  GSM972206  GSM972093  GSM972251  GSM972329  GSM972189  GSM972084  GSM972262  GSM972007  GSM972257  GSM972464  GSM972243  GSM971969  GSM972423  GSM972288  GSM972001  GSM972406  GSM972056  GSM972068  GSM972451  GSM972073  GSM972349  GSM972462  GSM972455  GSM972092  GSM972034  GSM972224  GSM972139  GSM972444  GSM972278  GSM972400  GSM972317  GSM972498  GSM972263  GSM972369  GSM972083  GSM972506  GSM972048  GSM1681359  GSM972050  GSM972438  GSM972442  GSM972199  GSM972222  GSM972032  GSM972094  GSM972005  GSM972424  GSM972255  GSM972244  GSM1681371  GSM972072  GSM972478  GSM972200  GSM972110  GSM972298  GSM972463  GSM972028  GSM972238  GSM972242  GSM972422  GSM972179  GSM1681363  GSM972311  GSM972074  GSM972250  GSM972428  GSM972248  GSM971979  GSM972283  GSM972205  GSM972474  GSM972316  GSM972294  GSM972115  GSM972232  GSM972022  GSM972472  GSM972507  GSM972129  GSM972234  GSM972292  GSM972024  GSM972389  GSM972063  GSM972078  GSM972501  GSM972450  GSM971999  GSM972228  GSM972038  GSM1681361  GSM972483  GSM972405  GSM972274  GSM972494  GSM972432  GSM972042  GSM972272  GSM972044  GSM972149  GSM972492  GSM972434  GSM972055  GSM972098  GSM972000  GSM972339  GSM972473  GSM972468  GSM972051  GSM972293  GSM972456  GSM972062  GSM972207  GSM972117  GSM972284  GSM972169  GSM972505  GSM972064  GSM1681369  GSM972282  GSM972006  GSM972399  GSM972401  GSM972023  GSM972233  GSM971989  GSM972273  GSM972268  GSM972493  GSM972256  GSM972407  GSM972310  GSM972484  GSM972482  GSM972057  GSM972201  GSM972088  GSM1681353  GSM972379  GSM972433  GSM972043  GSM972111  GSM972053  GSM972291  GSM972076  GSM972520  GSM972080  GSM972047  GSM972437  GSM972497  GSM972485  GSM972318  GSM972277  GSM972260  GSM972246  GSM972231  GSM972021  GSM972426  GSM972403  GSM972096  GSM972309  GSM972491  GSM972271  GSM972237  GSM972027  GSM972504  GSM972065  GSM972297  GSM972285  GSM972502  GSM972477  GSM972460  GSM972431  GSM972041  GSM972113  GSM972226  GSM972036  GSM972203  GSM1681365  GSM972281  GSM972008  GSM972090  GSM972045  GSM972159  GSM972054  GSM972435  GSM972402  GSM972495  GSM972052  GSM972487  GSM972404  GSM972275  GSM1681356  GSM972458  GSM972220  GSM972030  GSM972061  GSM972440  GSM972466  GSM972086  GSM972070  GSM972481  GSM972503  GSM972235  GSM972025  GSM972112  GSM972067  GSM972202  GSM972114  GSM972295  GSM972287  GSM972019  GSM972204  GSM972475  GSM972420  GSM972258  GSM972266  GSM972240  GSM972513  GSM972336  GSM972341  GSM972102  GSM1681364  GSM971990  GSM972459  GSM972212  GSM972135  GSM972104  GSM972009  GSM972396  GSM972187  GSM972214  GSM972195  GSM971961  GSM972327  GSM972158  GSM972166  GSM972140  GSM972365  GSM972018  GSM971967  GSM972181  GSM971970  GSM972321  GSM971986  GSM972259  GSM972412  GSM972175  GSM972353  GSM972414  GSM972380  GSM972376  GSM972120  GSM972347  GSM1681357  GSM972319  GSM972171  GSM971980  GSM972137  GSM971976  GSM972514  GSM972370  GSM972386  GSM972512  GSM972185  GSM972197  GSM972325  GSM972103  GSM972367  GSM972126  GSM972213  GSM972352  GSM971965  GSM972191  GSM971996  GSM972361  GSM972330  GSM972308  GSM972177  GSM972390  GSM972354  GSM972345  GSM972131  GSM972160  GSM972146  GSM972413  GSM972168  GSM971962  GSM972156  GSM972355  GSM1681360  GSM1681368  GSM972173  GSM972344  GSM972398  GSM972417  GSM972300  GSM972338  GSM972469  GSM971964  GSM972211  GSM972342  GSM972101  GSM972193  GSM972324  GSM972184  GSM972217  GSM972363  GSM972089  GSM972378  GSM972107  GSM971988  GSM972182  GSM972515  GSM972269  GSM972133  GSM972016  GSM972411  GSM971957  GSM972322  GSM972511  GSM972357  GSM972128  GSM972415  GSM972388  GSM972174  GSM972343  GSM972079  GSM972429  GSM972249  GSM971963  GSM971978  GSM972172  GSM972010  GSM972364  GSM972183  GSM972150  GSM972148  GSM972215  GSM972194  GSM972132  GSM972099  GSM972306  GSM972105  GSM972323  GSM971998  GSM972229  GSM972039  GSM972192  GSM972449  GSM972134  GSM972517  GSM972351  GSM972362 | GSE39582  GSE39582  GSE39582  GSE39582  GSE39582  GSE39582  GSE39582  GSE39582  GSE39582  GSE39582  GSE39582  GSE39582  GSE39582  GSE39582  GSE39582  GSE39582  GSE39582  GSE39582  GSE39582  GSE39582  GSE39582  GSE39582  GSE39582  GSE39582  GSE39582  GSE39582  GSE39582  GSE39582  GSE39582  GSE39582  GSE39582  GSE39582  GSE39582  GSE39582  GSE39582  GSE39582  GSE39582  GSE39582  GSE39582  GSE39582  GSE39582  GSE39582  GSE39582  GSE39582  GSE39582  GSE39582  GSE39582  GSE39582  GSE39582  GSE39582  GSE39582  GSE39582  GSE39582  GSE39582  GSE39582  GSE39582  GSE39582  GSE39582  GSE39582  GSE39582  GSE39582  GSE39582  GSE39582  GSE39582  GSE39582  GSE39582  GSE39582  GSE39582  GSE39582  GSE39582  GSE39582  GSE39582  GSE39582  GSE39582  GSE39582  GSE39582  GSE39582  GSE39582  GSE39582  GSE39582  GSE39582  GSE39582  GSE39582  GSE39582  GSE39582  GSE39582  GSE39582  GSE39582  GSE39582  GSE39582  GSE39582  GSE39582  GSE39582  GSE39582  GSE39582  GSE39582  GSE39582  GSE39582  GSE39582  GSE39582  GSE39582  GSE39582  GSE39582  GSE39582  GSE39582  GSE39582  GSE39582  GSE39582  GSE39582  GSE39582  GSE39582  GSE39582  GSE39582  GSE39582  GSE39582  GSE39582  GSE39582  GSE39582  GSE39582  GSE39582  GSE39582  GSE39582  GSE39582  GSE39582  GSE39582  GSE39582  GSE39582  GSE39582  GSE39582  GSE39582  GSE39582  GSE39582  GSE39582  GSE39582  GSE39582  GSE39582  GSE39582  GSE39582  GSE39582  GSE39582  GSE39582  GSE39582  GSE39582  GSE39582  GSE39582  GSE39582  GSE39582  GSE39582  GSE39582  GSE39582  GSE39582  GSE39582  GSE39582  GSE39582  GSE39582  GSE39582  GSE39582  GSE39582  GSE39582  GSE39582  GSE39582  GSE39582  GSE39582  GSE39582  GSE39582  GSE39582  GSE39582  GSE39582  GSE39582  GSE39582  GSE39582  GSE39582  GSE39582  GSE39582  GSE39582  GSE39582  GSE39582  GSE39582  GSE39582  GSE39582  GSE39582  GSE39582  GSE39582  GSE39582  GSE39582  GSE39582  GSE39582  GSE39582  GSE39582  GSE39582  GSE39582  GSE39582  GSE39582  GSE39582  GSE39582  GSE39582  GSE39582  GSE39582  GSE39582  GSE39582  GSE39582  GSE39582  GSE39582  GSE39582  GSE39582  GSE39582  GSE39582  GSE39582  GSE39582  GSE39582  GSE39582  GSE39582  GSE39582  GSE39582  GSE39582  GSE39582  GSE39582  GSE39582  GSE39582  GSE39582  GSE39582  GSE39582  GSE39582  GSE39582  GSE39582  GSE39582  GSE39582  GSE39582  GSE39582  GSE39582  GSE39582  GSE39582  GSE39582  GSE39582  GSE39582  GSE39582  GSE39582  GSE39582  GSE39582  GSE39582  GSE39582  GSE39582  GSE39582  GSE39582  GSE39582  GSE39582  GSE39582  GSE39582  GSE39582  GSE39582  GSE39582  GSE39582  GSE39582  GSE39582  GSE39582  GSE39582  GSE39582  GSE39582  GSE39582  GSE39582  GSE39582  GSE39582  GSE39582  GSE39582  GSE39582  GSE39582  GSE39582  GSE39582  GSE39582  GSE39582  GSE39582  GSE39582  GSE39582  GSE39582  GSE39582  GSE39582  GSE39582  GSE39582  GSE39582  GSE39582  GSE39582  GSE39582  GSE39582  GSE39582  GSE39582  GSE39582  GSE39582  GSE39582  GSE39582  GSE39582  GSE39582  GSE39582  GSE39582  GSE39582  GSE39582  GSE39582  GSE39582  GSE39582  GSE39582  GSE39582  GSE39582  GSE39582  GSE39582  GSE39582  GSE39582  GSE39582  GSE39582  GSE39582  GSE39582  GSE39582  GSE39582  GSE39582  GSE39582  GSE39582  GSE39582  GSE39582  GSE39582  GSE39582  GSE39582  GSE39582  GSE39582  GSE39582  GSE39582  GSE39582  GSE39582  GSE39582  GSE39582  GSE39582  GSE39582  GSE39582  GSE39582  GSE39582  GSE39582  GSE39582  GSE39582  GSE39582  GSE39582  GSE39582  GSE39582  GSE39582  GSE39582  GSE39582  GSE39582  GSE39582  GSE39582  GSE39582  GSE39582  GSE39582  GSE39582  GSE39582  GSE39582  GSE39582  GSE39582  GSE39582  GSE39582  GSE39582  GSE39582  GSE39582  GSE39582  GSE39582  GSE39582  GSE39582  GSE39582  GSE39582  GSE39582  GSE39582  GSE39582  GSE39582  GSE39582  GSE39582  GSE39582  GSE39582  GSE39582  GSE39582  GSE39582  GSE39582  GSE39582  GSE39582  GSE39582  GSE39582  GSE39582  GSE39582  GSE39582  GSE39582  GSE39582  GSE39582  GSE39582  GSE39582  GSE39582  GSE39582  GSE39582  GSE39582  GSE39582  GSE39582  GSE39582  GSE39582  GSE39582  GSE39582  GSE39582  GSE39582  GSE39582  GSE39582  GSE39582  GSE39582  GSE39582  GSE39582  GSE39582  GSE39582  GSE39582  GSE39582  GSE39582  GSE39582  GSE39582  GSE39582  GSE39582  GSE39582  GSE39582  GSE39582  GSE39582  GSE39582  GSE39582  GSE39582  GSE39582  GSE39582  GSE39582  GSE39582  GSE39582  GSE39582  GSE39582  GSE39582  GSE39582  GSE39582  GSE39582  GSE39582  GSE39582  GSE39582  GSE39582  GSE39582  GSE39582  GSE39582  GSE39582  GSE39582  GSE39582  GSE39582  GSE39582  GSE39582  GSE39582  GSE39582  GSE39582  GSE39582  GSE39582  GSE39582  GSE39582  GSE39582  GSE39582  GSE39582  GSE39582  GSE39582  GSE39582  GSE39582  GSE39582  GSE39582  GSE39582  GSE39582  GSE39582  GSE39582  GSE39582  GSE39582  GSE39582  GSE39582  GSE39582  GSE39582  GSE39582  GSE39582  GSE39582  GSE39582  GSE39582  GSE39582  GSE39582  GSE39582  GSE39582  GSE39582  GSE39582  GSE39582  GSE39582  GSE39582  GSE39582  GSE39582  GSE39582  GSE39582  GSE39582  GSE39582  GSE39582  GSE39582  GSE39582  GSE39582  GSE39582  GSE39582  GSE39582  GSE39582  GSE39582  GSE39582  GSE39582  GSE39582  GSE39582  GSE39582  GSE39582  GSE39582  GSE39582  GSE39582  GSE39582  GSE39582  GSE39582  GSE39582  GSE39582  GSE39582  GSE39582  GSE39582  GSE39582  GSE39582  GSE39582  GSE39582  GSE39582  GSE39582  GSE39582  GSE39582  GSE39582  GSE39582  GSE39582  GSE39582  GSE39582  GSE39582  GSE39582  GSE39582  GSE39582  GSE39582  GSE39582  GSE39582  GSE39582  GSE39582  GSE39582  GSE39582  GSE39582  GSE39582  GSE39582  GSE39582  GSE39582  GSE39582  GSE39582  GSE39582  GSE39582  GSE39582  GSE39582  GSE39582  GSE39582  GSE39582  GSE39582  GSE39582  GSE39582  GSE39582  GSE39582  GSE39582  GSE39582  GSE39582  GSE39582  GSE39582  GSE39582  GSE39582  GSE39582  GSE39582  GSE39582  GSE39582  GSE39582  GSE39582  GSE39582  GSE39582  GSE39582  GSE39582  GSE39582 | Low Immune Risk  Low Immune Risk  Low Immune Risk  Low Immune Risk  Low Immune Risk  Low Immune Risk  Low Immune Risk  High Immune Risk  Low Immune Risk  High Immune Risk  Low Immune Risk  Low Immune Risk  Low Immune Risk  Low Immune Risk  Low Immune Risk  Low Immune Risk  Low Immune Risk  Low Immune Risk  Low Immune Risk  Low Immune Risk  Low Immune Risk  Low Immune Risk  High Immune Risk  High Immune Risk  Low Immune Risk  Low Immune Risk  Low Immune Risk  Low Immune Risk  High Immune Risk  Low Immune Risk  Low Immune Risk  Low Immune Risk  High Immune Risk  Low Immune Risk  Low Immune Risk  Low Immune Risk  Low Immune Risk  Low Immune Risk  Low Immune Risk  Low Immune Risk  Low Immune Risk  Low Immune Risk  High Immune Risk  Low Immune Risk  High Immune Risk  Low Immune Risk  Low Immune Risk  High Immune Risk  Low Immune Risk  High Immune Risk  Low Immune Risk  Low Immune Risk  High Immune Risk  Low Immune Risk  Low Immune Risk  High Immune Risk  Low Immune Risk  High Immune Risk  Low Immune Risk  Low Immune Risk  High Immune Risk  High Immune Risk  High Immune Risk  Low Immune Risk  Low Immune Risk  Low Immune Risk  Low Immune Risk  High Immune Risk  Low Immune Risk  Low Immune Risk  Low Immune Risk  Low Immune Risk  High Immune Risk  Low Immune Risk  Low Immune Risk  Low Immune Risk  Low Immune Risk  Low Immune Risk  Low Immune Risk  Low Immune Risk  Low Immune Risk  High Immune Risk  Low Immune Risk  Low Immune Risk  Low Immune Risk  Low Immune Risk  Low Immune Risk  High Immune Risk  Low Immune Risk  Low Immune Risk  Low Immune Risk  Low Immune Risk  Low Immune Risk  Low Immune Risk  Low Immune Risk  High Immune Risk  High Immune Risk  Low Immune Risk  Low Immune Risk  Low Immune Risk  Low Immune Risk  High Immune Risk  Low Immune Risk  Low Immune Risk  High Immune Risk  Low Immune Risk  Low Immune Risk  Low Immune Risk  High Immune Risk  High Immune Risk  Low Immune Risk  High Immune Risk  Low Immune Risk  Low Immune Risk  Low Immune Risk  Low Immune Risk  Low Immune Risk  Low Immune Risk  High Immune Risk  High Immune Risk  Low Immune Risk  Low Immune Risk  High Immune Risk  Low Immune Risk  Low Immune Risk  Low Immune Risk  Low Immune Risk  Low Immune Risk  High Immune Risk  Low Immune Risk  Low Immune Risk  Low Immune Risk  Low Immune Risk  High Immune Risk  Low Immune Risk  Low Immune Risk  Low Immune Risk  Low Immune Risk  Low Immune Risk  High Immune Risk  Low Immune Risk  Low Immune Risk  Low Immune Risk  Low Immune Risk  Low Immune Risk  Low Immune Risk  Low Immune Risk  High Immune Risk  Low Immune Risk  Low Immune Risk  Low Immune Risk  High Immune Risk  Low Immune Risk  Low Immune Risk  High Immune Risk  Low Immune Risk  Low Immune Risk  Low Immune Risk  Low Immune Risk  Low Immune Risk  Low Immune Risk  High Immune Risk  Low Immune Risk  Low Immune Risk  Low Immune Risk  Low Immune Risk  Low Immune Risk  Low Immune Risk  Low Immune Risk  Low Immune Risk  Low Immune Risk  Low Immune Risk  High Immune Risk  Low Immune Risk  Low Immune Risk  High Immune Risk  Low Immune Risk  Low Immune Risk  High Immune Risk  High Immune Risk  High Immune Risk  High Immune Risk  Low Immune Risk  Low Immune Risk  High Immune Risk  Low Immune Risk  Low Immune Risk  High Immune Risk  Low Immune Risk  Low Immune Risk  Low Immune Risk  Low Immune Risk  Low Immune Risk  Low Immune Risk  Low Immune Risk  Low Immune Risk  High Immune Risk  Low Immune Risk  High Immune Risk  High Immune Risk  Low Immune Risk  Low Immune Risk  High Immune Risk  High Immune Risk  Low Immune Risk  High Immune Risk  Low Immune Risk  High Immune Risk  Low Immune Risk  Low Immune Risk  Low Immune Risk  Low Immune Risk  Low Immune Risk  Low Immune Risk  Low Immune Risk  Low Immune Risk  Low Immune Risk  Low Immune Risk  Low Immune Risk  Low Immune Risk  Low Immune Risk  Low Immune Risk  Low Immune Risk  Low Immune Risk  Low Immune Risk  Low Immune Risk  High Immune Risk  High Immune Risk  Low Immune Risk  High Immune Risk  Low Immune Risk  High Immune Risk  High Immune Risk  Low Immune Risk  High Immune Risk  Low Immune Risk  Low Immune Risk  Low Immune Risk  High Immune Risk  Low Immune Risk  Low Immune Risk  High Immune Risk  High Immune Risk  Low Immune Risk  High Immune Risk  High Immune Risk  High Immune Risk  Low Immune Risk  Low Immune Risk  Low Immune Risk  Low Immune Risk  Low Immune Risk  Low Immune Risk  Low Immune Risk  Low Immune Risk  Low Immune Risk  Low Immune Risk  Low Immune Risk  Low Immune Risk  High Immune Risk  Low Immune Risk  Low Immune Risk  Low Immune Risk  Low Immune Risk  High Immune Risk  Low Immune Risk  High Immune Risk  Low Immune Risk  Low Immune Risk  Low Immune Risk  Low Immune Risk  Low Immune Risk  High Immune Risk  High Immune Risk  Low Immune Risk  Low Immune Risk  Low Immune Risk  Low Immune Risk  Low Immune Risk  Low Immune Risk  Low Immune Risk  Low Immune Risk  High Immune Risk  Low Immune Risk  High Immune Risk  High Immune Risk  Low Immune Risk  Low Immune Risk  Low Immune Risk  Low Immune Risk  Low Immune Risk  High Immune Risk  Low Immune Risk  High Immune Risk  High Immune Risk  Low Immune Risk  Low Immune Risk  High Immune Risk  High Immune Risk  High Immune Risk  Low Immune Risk  Low Immune Risk  Low Immune Risk  Low Immune Risk  Low Immune Risk  Low Immune Risk  Low Immune Risk  Low Immune Risk  Low Immune Risk  Low Immune Risk  High Immune Risk  Low Immune Risk  Low Immune Risk  Low Immune Risk  Low Immune Risk  Low Immune Risk  High Immune Risk  Low Immune Risk  High Immune Risk  High Immune Risk  Low Immune Risk  Low Immune Risk  Low Immune Risk  Low Immune Risk  Low Immune Risk  Low Immune Risk  Low Immune Risk  Low Immune Risk  Low Immune Risk  High Immune Risk  High Immune Risk  Low Immune Risk  Low Immune Risk  Low Immune Risk  Low Immune Risk  Low Immune Risk  Low Immune Risk  Low Immune Risk  Low Immune Risk  Low Immune Risk  Low Immune Risk  Low Immune Risk  High Immune Risk  Low Immune Risk  Low Immune Risk  High Immune Risk  Low Immune Risk  High Immune Risk  High Immune Risk  Low Immune Risk  Low Immune Risk  Low Immune Risk  Low Immune Risk  Low Immune Risk  Low Immune Risk  Low Immune Risk  Low Immune Risk  Low Immune Risk  Low Immune Risk  High Immune Risk  Low Immune Risk  Low Immune Risk  Low Immune Risk  Low Immune Risk  Low Immune Risk  Low Immune Risk  Low Immune Risk  High Immune Risk  High Immune Risk  Low Immune Risk  Low Immune Risk  Low Immune Risk  Low Immune Risk  High Immune Risk  Low Immune Risk  Low Immune Risk  Low Immune Risk  Low Immune Risk  High Immune Risk  Low Immune Risk  Low Immune Risk  High Immune Risk  High Immune Risk  Low Immune Risk  Low Immune Risk  Low Immune Risk  Low Immune Risk  High Immune Risk  Low Immune Risk  Low Immune Risk  High Immune Risk  Low Immune Risk  Low Immune Risk  Low Immune Risk  Low Immune Risk  Low Immune Risk  High Immune Risk  Low Immune Risk  Low Immune Risk  High Immune Risk  Low Immune Risk  Low Immune Risk  High Immune Risk  Low Immune Risk  Low Immune Risk  High Immune Risk  Low Immune Risk  Low Immune Risk  Low Immune Risk  High Immune Risk  Low Immune Risk  High Immune Risk  High Immune Risk  Low Immune Risk  Low Immune Risk  Low Immune Risk  High Immune Risk  Low Immune Risk  High Immune Risk  Low Immune Risk  Low Immune Risk  High Immune Risk  Low Immune Risk  High Immune Risk  Low Immune Risk  Low Immune Risk  Low Immune Risk  Low Immune Risk  Low Immune Risk  Low Immune Risk  High Immune Risk  High Immune Risk  Low Immune Risk  Low Immune Risk  Low Immune Risk  Low Immune Risk  High Immune Risk  Low Immune Risk  Low Immune Risk  High Immune Risk  Low Immune Risk  Low Immune Risk  Low Immune Risk  Low Immune Risk  Low Immune Risk  High Immune Risk  Low Immune Risk  Low Immune Risk  Low Immune Risk  Low Immune Risk  Low Immune Risk  Low Immune Risk  Low Immune Risk  Low Immune Risk  Low Immune Risk  High Immune Risk  High Immune Risk  High Immune Risk  High Immune Risk  Low Immune Risk  High Immune Risk  Low Immune Risk  Low Immune Risk  High Immune Risk  Low Immune Risk  Low Immune Risk  High Immune Risk  Low Immune Risk  Low Immune Risk  High Immune Risk  High Immune Risk  Low Immune Risk  Low Immune Risk  Low Immune Risk  Low Immune Risk  High Immune Risk  High Immune Risk  Low Immune Risk  Low Immune Risk  Low Immune Risk  Low Immune Risk  High Immune Risk  Low Immune Risk  Low Immune Risk  Low Immune Risk  Low Immune Risk  Low Immune Risk  Low Immune Risk  Low Immune Risk  Low Immune Risk  Low Immune Risk  Low Immune Risk  High Immune Risk  High Immune Risk  High Immune Risk  High Immune Risk  High Immune Risk  Low Immune Risk  Low Immune Risk  High Immune Risk  High Immune Risk  Low Immune Risk  Low Immune Risk  High Immune Risk  High Immune Risk  High Immune Risk  High Immune Risk  High Immune Risk  Low Immune Risk  Low Immune Risk  High Immune Risk  Low Immune Risk  Low Immune Risk  Low Immune Risk  Low Immune Risk  Low Immune Risk  Low Immune Risk  Low Immune Risk  Low Immune Risk  Low Immune Risk  High Immune Risk  Low Immune Risk  Low Immune Risk  Low Immune Risk  Low Immune Risk  Low Immune Risk  Low Immune Risk  Low Immune Risk  Low Immune Risk  High Immune Risk  Low Immune Risk  Low Immune Risk  High Immune Risk  Low Immune Risk  Low Immune Risk  High Immune Risk  High Immune Risk  Low Immune Risk  Low Immune Risk  Low Immune Risk  Low Immune Risk  Low Immune Risk  Low Immune Risk  Low Immune Risk  Low Immune Risk  Low Immune Risk  Low Immune Risk  Low Immune Risk  Low Immune Risk  Low Immune Risk  Low Immune Risk  Low Immune Risk  Low Immune Risk  Low Immune Risk  Low Immune Risk  High Immune Risk  Low Immune Risk  High Immune Risk  Low Immune Risk  Low Immune Risk  Low Immune Risk  Low Immune Risk  Low Immune Risk  Low Immune Risk  Low Immune Risk  Low Immune Risk  Low Immune Risk  Low Immune Risk  High Immune Risk  Low Immune Risk  Low Immune Risk  High Immune Risk  High Immune Risk  High Immune Risk  High Immune Risk  Low Immune Risk  Low Immune Risk  Low Immune Risk  Low Immune Risk | -1.557982685  -0.77150904  -1.322789748  -0.880848725  -0.897288001  -1.517291791  -1.643262116  0.647684906  -2.73472666  -0.364470859  -0.491256011  -0.622159528  -2.107782786  -1.330097356  -1.272778832  -0.754434127  -0.931038811  -2.364122645  -0.470842826  -2.288239026  -0.987289951  -0.896374788  0.388963477  0.086659676  -1.168725013  -1.104873699  -1.093150548  -0.918204423  0.243445731  -1.359949344  -0.732149412  -1.921639212  0.321956366  -0.633939876  -0.931038811  -1.050867387  -2.505087461  -1.444785186  -1.553330607  -0.786723983  -0.673554492  -1.442923398  0.592934289  -1.778249227  -0.459413996  -0.724514245  -1.354868587  -0.34678518  -1.528291393  -0.146031766  -1.3764337  -1.475151578  -0.312989009  -2.683570197  -2.363052888  -0.348918611  -1.613425415  0.667546205  -1.485871916  -1.711617674  -0.415052176  -0.181100849  -0.235793223  -1.878143587  -0.885338434  -0.73615291  -1.93829827  0.724740116  -0.688739852  -2.156520682  -2.053357556  -0.641271303  -0.33263052  -1.636480299  -1.27808802  -2.711619667  -1.539638182  -1.311442958  -0.953044019  -3.041153226  -1.261017453  0.043140792  -1.755978139  -1.147709504  -1.506812215  -0.651268  -1.850094117  0.09943447  -0.620799964  -1.612557256  -0.682903178  -1.850094117  -1.360583052  -0.976913331  -1.33418016  -0.332680017  0.910558303  -0.918730125  -1.818058901  -1.790009431  -2.477037991  -0.333677436  -0.815759343  -0.858119737  0.401821254  -1.019089322  -1.617352356  -1.207451197  -0.334926036  -0.1895527  -0.917548352  0.604212897  -1.350839217  -0.844768815  -0.894808153  -2.555242688  -1.795668887  -0.917619994  0.424924509  -0.317356142  -2.056626323  -0.611873163  -0.201488503  -1.478103791  -1.107291517  -1.77498046  -1.641424601  -1.144609146  0.096151552  -1.04199605  -2.73472666  -1.362354109  -2.505087461  0.510296242  -2.156520682  -1.926247807  -1.95704123  -0.467133482  -1.511632335  -0.182992069  -0.541956384  -1.911069495  -1.400083458  -2.294787858  -1.042380855  -0.981049866  -0.640860454  0.216642802  -2.053357556  -1.071407452  -0.620641959  0.906495046  -1.071214538  -0.473258393  -0.101586643  -0.855560253  -1.130355084  -1.770044783  -1.447164167  -1.272778832  -1.555238731  -0.437615938  -0.97043507  -1.485871916  -0.93103895  -2.203121131  -1.016406805  -0.558959922  -2.135832256  -1.431022277  -1.500241923  -2.456349564  -0.236835319  -0.992440279  -0.804221286  -0.282807054  -1.200667923  -1.528291393  0.55532294  0.284475927  -0.377894889  -0.446210668  -0.91427096  -1.713976117  0.327796311  -1.643350679  -2.416953305  -0.128056066  -0.882851085  -0.663276511  -1.612557256  -1.803645271  -1.703435365  -0.834871826  -0.909750928  -0.631989173  0.097559417  -1.935125711  0.322776058  -0.201789276  -1.242553254  -1.82940569  -0.030042526  -0.448708055  -1.664039105  -0.396864266  -2.73472666  -0.028499471  -2.096435996  -1.560326609  -0.847077113  -2.159264636  -2.23950113  -1.818058901  -1.671356526  -1.375345921  -1.321290315  -2.494771232  -0.641271303  -1.037095231  -2.757840453  -1.790009431  -0.65362593  -2.989996762  -2.188344667  -1.095805939  0.483891795  -0.20785931  -1.882451212  0.015734151  -2.486288092  -0.39044243  0.30316678  -0.786723983  -0.134652742  -1.681744668  -1.44692074  -2.320832945  0.07785049  -2.267550599  -0.726639297  -0.117122723  0.351096267  -1.058105076  0.19157785  1.037975487  0.004784346  -0.692832827  -1.250533377  -1.33418016  -0.936494897  -1.618067371  -0.82455117  -0.509791002  -1.33418016  -0.638241036  -1.77498046  -1.871104422  -1.235937756  -0.14072187  -0.515286971  -0.574956305  -2.456349564  -2.053357556  -0.45553279  -0.996067938  -0.00924143  -1.511632335  -1.2094019  -0.936753276  -1.329244483  -1.485212952  -0.066825973  -0.19179929  -3.041153226  -1.300294155  -1.961124034  -1.33418016  -2.465691201  -1.790009431  -2.558132982  -2.00986193  -0.406433162  -1.182347828  0.856133073  0.441101956  -2.783464556  -1.10234918  -2.107782786  -1.272394026  -0.97803682  -0.443326524  -0.545104318  0.353276568  -0.398972344  -1.428971471  -1.500241923  0.159683058  -0.286850574  0.592784948  -1.490961567  -1.120075818  -1.039520598  -1.625133509  -0.966971553  -1.32283337  -2.23950113  -1.790009431  -1.482319658  -0.779727981  0.222461415  -2.73472666  -2.76277613  -1.918983821  -1.113248922  -2.505087461  -0.309591045  -0.918164401  -0.203364436  -0.070096432  -1.578657105  -2.096435996  -0.793178718  -1.401512658  -1.33418016  -0.654853708  -1.136646174  -0.964176158  -1.148036586  -0.008473478  0.040261149  -2.216394136  -1.485828293  -0.521999289  -0.70349302  -1.056617939  -2.198660895  -1.621301987  -1.745134097  -0.987190034  -2.73472666  -0.993256189  0.121789806  -1.684635997  -0.726639297  0.220704364  -0.563360258  0.559034138  -0.25718295  -1.276287467  -1.428304902  -1.354868587  -1.487665809  -0.50341121  -1.212090984  -0.753139379  -0.85318406  -0.97803682  -0.641257296  0.164532473  -0.862072261  -1.776869519  -2.732308093  -1.371527644  -0.926880356  -1.689612466  -1.608595633  -0.455127729  -0.213731081  -0.757017076  -1.83213183  -1.790009431  -0.544006272  0.902104807  -0.863779193  -0.842807111  -1.061839952  -1.921727775  -0.394363749  -1.010437945  -1.999714881  0.080938036  0.064901493  -0.545104318  -1.136646174  -0.516529197  -1.175893092  0.080938036  -1.418019105  -0.702373489  -0.296131604  -2.456349564  -0.750573589  -1.539638182  -0.488793822  -0.997257032  -0.219038323  -1.935125711  -1.249829666  0.461304507  -2.107782786  -1.018209462  -0.141948962  -2.477037991  -1.619384153  0.388257357  -1.921727775  -1.33418016  -2.672223407  0.054219598  -2.193725218  0.815908179  -0.155005576  -1.506696659  -2.416953305  -2.989996762  -0.023880465  -2.486288092  -0.313647973  -2.465691201  -0.526562805  0.048124166  -1.040329848  0.043972346  -1.036336595  -2.290894417  -0.524158251  -1.34101366  -0.911927036  -1.78329059  0.229189199  0.006601578  -3.041153226  -0.493131945  -1.041185676  -1.224590966  0.36599542  -1.132771629  -1.850094117  0.17648968  -0.485038986  -0.818872819  -0.472847947  -0.772901084  -1.878476325  0.449266519  -1.441789239  -2.783464556  -1.732690907  -0.740331721  -2.414209352  -0.723361197  -0.514133265  -0.853333401  -1.168725013  0.115739707  -0.415939729  0.056200166  -0.383987695  -2.589454219  0.287988469  -1.041274045  -0.945681498  0.321956366  -1.161960174  -2.107782786  0.851831913  -0.468950628  -1.549648886  0.045328771  0.169128038  -0.993301776  -0.604389359  -0.46656322  -2.107782786  0.939088965  0.266480275  -1.240195323  -0.518887639  -2.783464556  -0.468950628  -0.21267943  -1.921727775  -1.33418016  -1.04279504  -2.73472666  -2.23519997  -1.062743148  -1.711617674  -0.650401116  -1.794092374  -1.29478404  0.63696356  -0.17892862  -0.091839414  -0.107097736  -0.332948275  -0.956251215  -1.271633285  0.592934289  -0.210260863  -1.08534037  -1.440096004  0.864871933  -0.278116927  0.261857674  -0.434507049  0.472651297  -1.386188789  -1.766858815  -0.313915731  -1.608474452  -0.560105  -2.451413888  -0.883095318  -2.732308093  -2.783464556  -1.233979018  -1.107534944  -2.228110857  -0.143129816  -2.107782786  -0.60119575  -0.515172393  -0.772901084  -1.320668641  -0.546950458  -2.062907452  -0.906773003  0.689839269  -0.97803682  -0.619526246  -0.337570876  -0.607468778  -0.650159503  -0.347815881  -0.452097462  -1.961124034  -1.790009431  -1.601210466  -1.738852968  -1.040329848  -1.87575604  -0.803645152  -0.839806795  -2.451413888  -1.931189108  -1.80408236  -1.062668592  -0.697273185  -1.640606725  -1.353325531  -0.609692862  -1.125507643  -1.640072579  0.047376832  -0.749503832  0.266310266  -1.305967481  -1.82940569  -1.426413682  -1.77498046  -0.793008709  -0.519105995  -0.476027554  -0.863779193  -0.890347917  -1.142156289  0.130787044  -1.512356114  -2.288239026  -0.081198989  0.118233838  0.039693719  -0.065795272  -2.053357556  -1.748650237  -0.878957505  -0.487206568 |
