# Supplementary material for: Identification and Verification of a 17 Immune-Related Gene Pair Prognostic Signature for Colon Cancer
Source: Biomed Res Int. 2021 May 22;2021:6057948. doi: 10.1155/2021/6057948 (PMC8166469; doi:10.1155/2021/6057948)
Supplement: Supplementary Materials — Supplementary Table 1: Patients' immune risk stratification. Supplementary Table 2: GSEA. [file 6057948.f1.zip › Table S2 GSEA.docx]

Table S2. GSEA

| **Gene.Set.Term** | **ES** | **NES** | ***P*-value** |
| --- | --- | --- | --- |
| KEGG_CITRATE_CYCLE_TCA_CYCLE  KEGG_RNA_DEGRADATION  KEGG_PEROXISOME  KEGG_RIBOSOME  KEGG_FOCAL_ADHESION  KEGG_NEUROACTIVE_LIGAND_RECEPTOR_INTERACTION  KEGG_OLFACTORY_TRANSDUCTION  KEGG_CALCIUM_SIGNALING_PATHWAY  KEGG_CELL_ADHESION_MOLECULES_CAMS  KEGG_ECM_RECEPTOR_INTERACTION  KEGG_SYSTEMIC_LUPUS_ERYTHEMATOSUS | -0.686244786  -0.550526406  -0.498967435  -0.468371146  0.486679724  0.492214329  0.495375534  0.49677384  0.506573182  0.585789548  0.593868841 | -1.626146086  -1.466464938  -1.405329962  -1.347201048  1.360041415  1.413795349  1.461819583  1.373984867  1.352250581  1.479156595  1.41506215 | 0.014227  0.028341498  0.03016158  0.041031227  0.019593346  0.003330558  0.000569995  0.020989505  0.035183737  0.012164777  0.036047498 |
